# Investing in the future: addressing the rising cost of perfusion education in 2025

**DOI:** 10.1051/ject/2025029

**Published:** 2025-09-15

**Authors:** Blaine Johnson

**Affiliations:** 1 Perfusion Services, UChicago Medicine 5841 S Maryland Ave, Ste E500, MC5040 Chicago IL 60637 USA; 2 Perfusion Education Program, University of Iowa Carver College of Medicine 200 Hawkins Dr, C43-Z GH Iowa City IA 52242 USA

**Keywords:** Perfusion Education Program, Medical Education, Costs and Cost Analysis, Financial Aid, Scholarship

## Abstract

The demand for allied healthcare professionals has surged, raising concerns about the rising costs of education. Tuition for post-baccalaureate and master’s programs in perfusion technology ranges from $18,000 to $106,500 annually, often surpassing $100,000 in total expenses. This financial burden presents significant challenges for prospective students, restricting their entry into the field. High costs could lead to a reduction in the number of qualified perfusionists, negatively impacting patient care. To address these challenges, partnerships between academic institutions and healthcare organizations could facilitate the development of scholarships or sponsored work studies. Additionally, policymakers should advocate for increased funding and other initiatives to help alleviate the financial strain allied health professionals face. Creating innovative solutions to these financial challenges may lead to a more diverse group of professionals in the field, enriching perspectives and approaches to patient care. Investing in accessible education will strengthen the healthcare system, benefiting providers and patients.

## Discussion

As the demand for allied healthcare professionals continues to grow, addressing the rising costs of education in specialized fields such as perfusion technology has become increasingly urgent. The financial burden associated with pursuing a career in this specialty has grown substantially, raising concerns among prospective students and within the profession. Previous work has highlighted the rapid expansion of Perfusion Education Programs (PEPs) and emphasized the need for efficient application and educational pathways to support the field’s growth and sustainability in the U.S. [1–4].

Over the past decade, higher education costs in the United States have risen in nearly every discipline, particularly in allied healthcare training programs. This increase is partly due to inflation, higher operational expenses, and a greater reliance on tuition as a primary revenue source [5, 6]. High costs are associated with low student-to-faculty ratios and the need for extensive clinical facilities. Tuition fees for a post-baccalaureate certificate or master’s degree in perfusion technology range from $18,000 to $106,500 per year, depending on the institution (Table 1). These figures do not include additional expenses such as clinical rotation fees, textbooks, supplies, and living costs, which can easily push the total cost of education beyond $100,000.

**Table 1:** Comparison of degree type and tuition cost for perfusion education programs in the United States.

Sponsor	City, State	Degree type	Program length	Total cost*	Cost per month
Baylor Scott & White The Heart Hospital at Plano	Plano, TX	Certificate	12-month	$32,000	$2,666.67
University of Texas Health Science Center at Houston	Houston, TX	Certificate	12-month	$18,000	$1,500.00
Cleveland Clinic Foundation	Cleveland, OH	Certificate	17-month	$32,000	$1,882.35
Texas Heart Institute	Houston, TX	Certificate	18-month	$30,000	$1,666.67
University of Iowa	Iowa City, IA	Certificate	21-month	$24,350 I	$1,159.52 I
				$79,258 O	$3,774.19 O
Vanderbilt University Medical Center	Nashville, TN	Certificate	22-month	$39,000	$1,772.73
UPMC Presbyterian Shadyside	Pittsburgh, PA	Masters	18-month	$71,520	$3,973.33
SUNY Upstate Medical University	Syracuse, NY	Masters	19-month	$28,275 I	$1,488.16 I
				$57,750 O	$3,039.47 O
Keck School of Medicine of USC	Los Angeles, CA	Masters	20-month	$141,064	$7,053.20
Emory University	Atlanta, GA	Masters	21-month	$106,437	$5,068.45
Hofstra University	Hempstead, NY	Masters	21-month	$105,090	$5,004.29
Lawrence Technological University	Southfield, MI	Masters	21-month	$92,530	$4,406.19
Midwestern University	Glendale, AZ	Masters	21-month	$99,192	$4,723.43
Rush University	Chicago, IL	Masters	21-month	$82,758	$3,940.86
Thomas Jefferson University	Philadelphia, PA	Masters	21-month	$37,500	$1,785.71
University of Nebraska Medical Center	Omaha, NE	Masters	21-month	$18,320 I	$872.38 I
				$45,420 O	$2,162.86 O
Lipscomb University	Nashville, TN	Masters	22-month	$87,000	$3,954.44
Medical University of South Carolina	Charleston, SC	Masters	22-month	$40,745 I	$1,852.05 I
				$64,155 O	$2,916.14 O
Milwaukee School of Engineering	Milwaukee, WI	Masters	24-month	$43,800	$1,825.00
Northern Kentucky University	Highland Heights, KY	Masters	24-month	$61,320	$2,555.00
Quinnipiac University	Hamden, CT	Masters	24-month	$58,395	$2,433.13
University of Arizona	Tucson, AZ	Masters	24-month	$32,875 I	$1,369.79 I
				$72,760 O	$3,031.67 O
University of Utah	Salt Lake City, UT	Masters	24-month	$21,689 I	$903.71 I
				$76,577 O	$3,190.72 O
University of Texas Health Science Center at Tyler	Tyler, TX	Masters	PENDING	PENDING	PENDING
Virginia Commonwealth University	Richmond, VA	Masters	PENDING	PENDING	PENDING

*Cost of Tuition was based on data for the 2024-2025 academic year available on January 1, 2025.

I, In-state Tuition; O, Out-of-state Tuition.

The accumulation of student loan debt poses a significant concern for applicants, as it can take many years to repay, potentially limiting their ability to invest in their careers or pursue further professional development [7]. These high costs have broader implications. If fewer students can fund their essential training, there may be a lack of qualified perfusionists, ultimately affecting patient care throughout the United States [8, 9]. With the healthcare system already experiencing workforce deficits across multiple specialties, failure to address rising educational costs could deepen these challenges [10, 11].

Currently, aspiring professionals can choose between two entry-level degrees: a post-baccalaureate certificate (24% [6/25]) or a master’s degree (76% [19/25]). This decision can significantly influence both the cost and duration of their educational journey. While some have proposed making a master’s degree the standard entry-level credential, post-baccalaureate certificate programs offered by universities and hospitals in the United States present practical alternatives for those seeking a faster and more affordable pathway to education in this field [12–14]. Master’s programs typically demand more time and higher tuition, while certificate programs often provide a quicker and more affordable option.

While certificate programs can be completed in as little as 12 months, master’s programs typically require 18–24 months. This variation in time commitment affects the overall costs of education and how quickly graduates can enter the workforce. The cost of perfusion education also varies based on whether an institution is state-funded or private. State-funded programs usually offer lower tuition rates for in-state students, while private institutions often impose higher fees. Prospective students should consider these differences when evaluating their options. Geographic location also influences the cost and availability of perfusion education programs. Some regions have more accredited programs and a higher demand for perfusionists, which in turn impacts tuition rates and job opportunities. Prospective students must account for these factors when deciding where to pursue their education.

In the context of federal student aid for PEPs, the type of loan awarded is primarily determined by the degree the program offers. Students enrolled in post-baccalaureate certificate programs are considered undergraduate borrowers for federal aid purposes. Consequently, they may qualify for Direct Subsidized Loans and Direct Unsubsidized Loans, provided they have not reached their aggregate loan limits for undergraduate borrowing. In contrast, students in master’s degree programs are classified as graduate-level borrowers. They are only eligible for Direct Unsubsidized Loans or Graduate PLUS Loans, the latter of which requires a credit check, has higher interest rates, and may necessitate a parental co-signer.

Regardless of the program type, most PEP programs require applicants to have obtained a bachelor’s degree before matriculation. As a result, they are ineligible for Federal Pell Grants or Federal Supplemental Educational Opportunity Grants, both of which are designated for undergraduate students pursuing their first degree. It is also important to note that not all PEPs offer federal student aid. Some hospital-based, non-credit-bearing programs may not be recognized as institutions of higher education by the Department of Education. These programs may offer only private loans or no loan options at all.

To address these issues, institutions could explore partnerships with hospitals and healthcare organizations to subsidize tuition or offer scholarships. Models such as employer-sponsored scholarships, loan repayment assistance, and service-based tuition reimbursement programs not only alleviate the financial burden on students but also encourage long-term employment commitments. These approaches have proven effective in fields like nursing and primary care and could be adapted to perfusion education, particularly in underserved areas where workforce shortages are most severe [15–17].

Expanding financial aid options and offering more affordable tuition would also increase accessibility for a broader range of students. Targeted scholarship programs for those pursuing perfusion technology could be a strategic way to enhance and support diversity [18, 19]. Additionally, building strong alumni networks can improve the educational experience by providing mentorship, career guidance, and financial assistance to current students [20].

While these strategies lay a strong foundation, sustained change will require active policy engagement. Policymakers must build on these efforts by advocating for increased funding of allied health education programs and promoting policies that reduce financial barriers to entry. Federal and state legislators must recognize perfusionists as an essential component of the allied health workforce.

## Conclusion

The rising cost of education in perfusion technology represents a considerable barrier for aspiring professionals and threatens the sustainability of the workforce at a time when the demand for perfusionists is increasing. With total educational expenses often surpassing $100,000, many students encounter significant debt burdens that may discourage them from entering the field or restrict their long-term career development. By promoting collaborations between educational institutions and healthcare organizations, improving financial aid options, and advocating for increased funding, we can ease the financial burdens facing prospective students. Together, we can create a more accessible and sustainable educational environment, benefiting healthcare systems and patients.
