# Corrigendum to: Investing in the future: addressing the rising cost of perfusion education in 2025

**DOI:** 10.1051/ject/2026024

**Published:** 2026-09-15

**Authors:** Blaine Johnson

**Affiliations:** 1 Perfusion Services, UChicago Medicine 5841 S Maryland Ave, Ste E500, MC5040 Chicago IL 60637 USA; 2 Perfusion Education Program, University of Iowa Carver College of Medicine 200 Hawkins Dr, C43-Z GH Iowa City IA 52242 USA

In the originally published version of Table 1, several tuition values were reported incorrectly. Specifically, the total tuition and corresponding cost-per-month figures for multiple programs, including SUNY Upstate Medical University, Thomas Jefferson University, the University of Nebraska Medical Center, and the Milwaukee School of Engineering, were inaccurate. These discrepancies were due to errors in transcription and/or calculation.

**Table 1:** Comparison of Degree Type and Tuition Cost for Perfusion Education Programs in the United States.

Sponsor	City, State	Degree Type	Program Length	Total Cost*	Cost per Month
Baylor Scott & White The Heart Hospital at Plano	Plano, TX	Certificate	12-month	$32,000	$2,666.67
University of Texas Health Science Center at Houston	Houston, TX	Certificate	12-month	$18,000	$1,500.00
Cleveland Clinic Foundation	Cleveland, OH	Certificate	17-month	$32,000	$1,882.35
Texas Heart Institute	Houston, TX	Certificate	18-month	$30,000	$1,666.67
University of Iowa	Iowa City, IA	Certificate	21-month	$24,350 I	$1,159.52 I
				$79,258 O	$3,774.19 O
Vanderbilt University Medical Center	Nashville, TN	Certificate	22-month	$39,000	$1,772.73
UPMC Presbyterian Shadyside	Pittsburgh, PA	Masters	18-month	$71,520	$3,973.33
SUNY Upstate Medical University	Syracuse, NY	Masters	19-month	$28,275 I	$1,488.16 I
				$62,475 O	$3,288.15 O
Keck School of Medicine of USC	Los Angeles, CA	Masters	20-month	$141,064	$7,053.20
Emory University	Atlanta, GA	Masters	21-month	$106,437	$5,068.45
Hofstra University	Hempstead, NY	Masters	21-month	$105,090	$5,004.29
Lawrence Technological University	Southfield, MI	Masters	21-month	$92,530	$4,406.19
Midwestern University	Glendale, AZ	Masters	21-month	$99,192	$4,723.43
Rush University	Chicago, IL	Masters	21-month	$82,758	$3,940.86
Thomas Jefferson University	Philadelphia, PA	Masters	21-month	$86,520	$4,120.00
University of Nebraska Medical Center	Omaha, NE	Masters	21-month	$32,620 I	$1,553.33 I
				$82,580 O	$3,932.38 O
Lipscomb University	Nashville, TN	Masters	22-month	$87,000	$3,954.44
Medical University of South Carolina	Charleston, SC	Masters	22-month	$40,745 I	$1,852.05 I
				$64,155 O	$2,916.14 O
Milwaukee School of Engineering	Milwaukee, WI	Masters	24-month	$67,160	$2,798.33
Northern Kentucky University	Highland Heights, KY	Masters	24-month	$61,320	$2,555.00
Quinnipiac University	Hamden, CT	Masters	24-month	$58,395	$2,433.13
University of Arizona	Tucson, AZ	Masters	24-month	$32,875 I	$1,369.79 I
				$72,760 O	$3,031.67 O
University of Utah	Salt Lake City, UT	Masters	24-month	$21,689 I	$903.71 I
				$76,577 O	$3,190.72 O
University of Texas Health Science Center at Tyler	Tyler, TX	Masters	PENDING	PENDING	PENDING
Virginia Commonwealth University	Richmond, VA	Masters	PENDING	PENDING	PENDING

*Cost of Tuition was based on data for the 2024-2025 academic year available on January 1, 2025.

I, In-state Tuition; O, Out-of-state Tuition.

The tuition values have been updated to accurately reflect both in-state and out-of-state costs where applicable, and the corresponding monthly cost calculations have been revised accordingly. These corrections do not affect the overall comparisons or conclusions presented in the table.

The author regrets these errors and any confusion they may have caused.

